# Glutathione and the Antioxidant Potential of Binary Mixtures with Flavonoids: Synergisms and Antagonisms

**DOI:** 10.3390/molecules18088858

**Published:** 2013-07-25

**Authors:** Renato B. Pereira, Carla Sousa, Andreia Costa, Paula B. Andrade, Patrícia Valentão

**Affiliations:** REQUIMTE/Laboratório de Farmacognosia, Departamento de Química, Faculdade de Farmácia, Universidade do Porto, Rua de Jorge Viterbo Ferreira, nº 228, 4050-313 Porto, Portugal; E-Mails: ren.pereira@gmail.com (R.B.P.); carlasarasousa70@gmail.com (C.S.); andreiadfcosta@gmail.com (A.C.); pandrade@ff.up.pt (P.B.A.)

**Keywords:** flavonoids, glutathione, antioxidant activity, synergism, antagonism

## Abstract

Polyphenols are able to trap free radicals, which contributes to their known antioxidant capacity. In plant extracts, these secondary metabolites may act in concert, in a way that their combined activities will be superior to their individual effects (synergistic interaction). Several polyphenols have demonstrated clear antioxidant properties *in vitro*, and many of their biological actions have been attributed to their intrinsic reducing capabilities. As so, the intake of these compounds at certain concentrations in the diet and/or supplementation may potentiate the activity of reduced form glutathione (GSH), thus better fighting oxidative stress. The aim of this work was to predict a structure-antioxidant activity relationship using different classes of flavonoids and to assess, for the first time, possible synergisms and antagonisms with GSH. For these purposes a screening microassay involving the scavenging of DPPH^•^ was applied. In general, among the tested compounds, those lacking the catechol group in B ring showed antagonistic behaviour with GSH. Myricetin displayed additive effect, while quercetin, fisetin, luteolin, luteolin-7-*O*-glucoside, taxifolin and (+)-catechin demonstrated synergistic actions. Furthermore, adducts formed at C2′ and C5′ of the B ring seem to be more important for the antioxidant capacity than adducts formed at C6 and C8 of the A ring.

## 1. Introduction

The most important endogenous antioxidant defence systems are composed of the thiol-containing tripeptide glutathione and small thiol-containing proteins, such as thioredoxin, glutaredoxin, and peroxiredoxin. Of these, glutathione is found at millimolar concentrations in most cells and is the major contributor to the cell’s redox state. Glutathione occurs in cells in both reduced (GSH) and oxidized (GSSG) forms. It may also covalently bind to proteins through glutathionylation [1,2]. One important task of cellular glutathione is to scavenge free radicals and peroxides produced during normal cellular respiration, which would otherwise oxidize proteins, lipids and nucleic acids [3,4].

Phenolic compounds, characterized by hydroxylated aromatic rings, are ubiquitous in plants. Simple phenolics and flavonoids are important constituents of plants, showing a wide range of antioxidant activities *in vitro* [5]. Flavonoids, in particular, have characteristics that make them the phenolic compounds with the strongest antioxidant capacity [6]. In contrast to GSH, flavonoids cannot be synthesized by humans, but are obtained through the diet; so, free radicals that are originated during body metabolism and their presence in blood can be neutralized by regular intake of foods containing a high content of these antioxidants, such as fruits and vegetables [7]. For flavonoids, in general it is assumed that they are absorbed as their aglycones after prior hydrolysis of the glycosides along the digestive tract. Small amounts of aglycones can also be present in the diet. Moreover, the health food industry is now providing increasing numbers of flavonoid aglycones as food supplements [8]. Classified as chain breaking antioxidants, these flavonoid aglycones are reported to quench free radicals by donating a hydrogen atom and/or an electron to free radicals [9]. Many studies revealed that the mentioned properties bear preventive potential against several degenerative diseases, such as cancer or cardiovascular diseases [10,11].

Chemical reactions involving the transfer of an electron and a proton can occur by means of concerted or stepwise mechanisms. The position and degree of hydroxylation, polarity, solubility and reducing potential are the main factors influencing the antioxidant activity of phenolic compounds [12,13,14]. In most methods used to evaluate antioxidant properties, the ability of the antioxidants to trap free radicals is assessed by the kinetic of their reactions with a radical. The methods applying chromogenic compounds are commonly used due to their ease, speed and sensitivity, the most popular being those employing the stable 2,2-diphenyl-1-picrylhydrazyl radical (DPPH^•^) and 2,2-azinobis(3-ethyl-benzothiazoline-6-sulphonicacid) cation radical (ABTS^•+^) [15,16].

For this study flavonoids were considered. This type of molecules comprises a broad collection of plant metabolites possessing a C_6_-C_3_-C_6_ skeleton, or, more specifically, a phenylbenzopyran function. The typical flavone ring is the backbone of the flavonoid structure, or the nucleus of diverse flavonoid molecules [17].

The scavenging of free radicals by flavonoids often involves the formation of a phenoxyl radical that after is converted to a quinone. For example, quercetin (Table 1) is oxidized to a quinone when serving as an antioxidant, and this quinone reacts with thiols [18]. On the other hand, the chemical properties of some antioxidants may also confer them with pro-oxidant properties and this should be considered with respect to mechanisms for induction of cellular antioxidant defences. Flavonoids can auto-oxidize and products of auto-oxidation can possibly react with or otherwise reduce cellular concentrations of glutathione [19].

**Table 1:** Structures of the studied flavonoids.

Compound	Structure
Quercetin	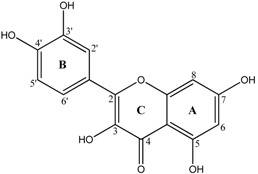
Quercetagetin	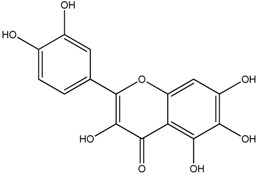
Isorhamnetin	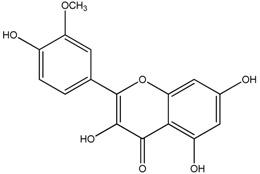
Fisetin	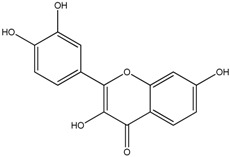
Galangin	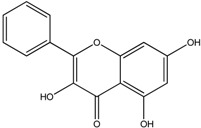
Myricetin	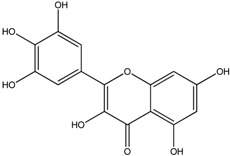
Luteolin	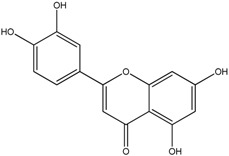
Luteolin-7-*O*-glucoside	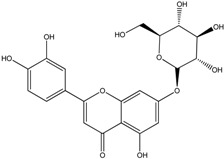
Taxifolin	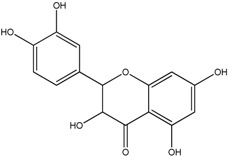
(+)-Catechin	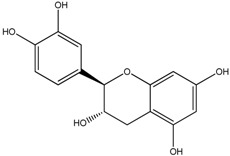

To shed some light on the knowledge about the behaviour of these exogenous antioxidants when in the presence of this endogenous antioxidant, in the present work several classes of flavonoids were mixed with GSH and the resulting DPPH^•^ scavenging ability was assessed for the first time to find synergistic or antagonistic interactions.

## 2. Results and Discussion

### 2.1. DPPH^•^ Scavenging Activity of Flavonoids

The antioxidant mechanisms of flavonoids comprise free radical scavenging, but also enzyme inhibition/induction, metal chelation, interaction with receptors and modulation of gene expression [20]. The direct scavenging of radicals is believed to occur in cells, but, to avoid the interference of other mechanisms, this work was developed using a cell-free system.

The past few decades of structure-activity relationships research have generated several consistent lines of evidence supporting the role of specific structural components as requisites for antioxidant activity. Some characteristics of flavonoids are important to the antioxidant capacity: *o*-dihydroxy structure in the B ring, which confers higher stability to the radical form and participates in electron delocalization [21]; C2=C3 double bond in conjugation with a 4-oxo function in the C ring, responsible for electron delocalization of the aromatic nucleus; 3- and 5-OH groups with 4-oxo function in the A and C rings, required for maximum radical scavenging potential [22,23,24].

**Figure 1:**
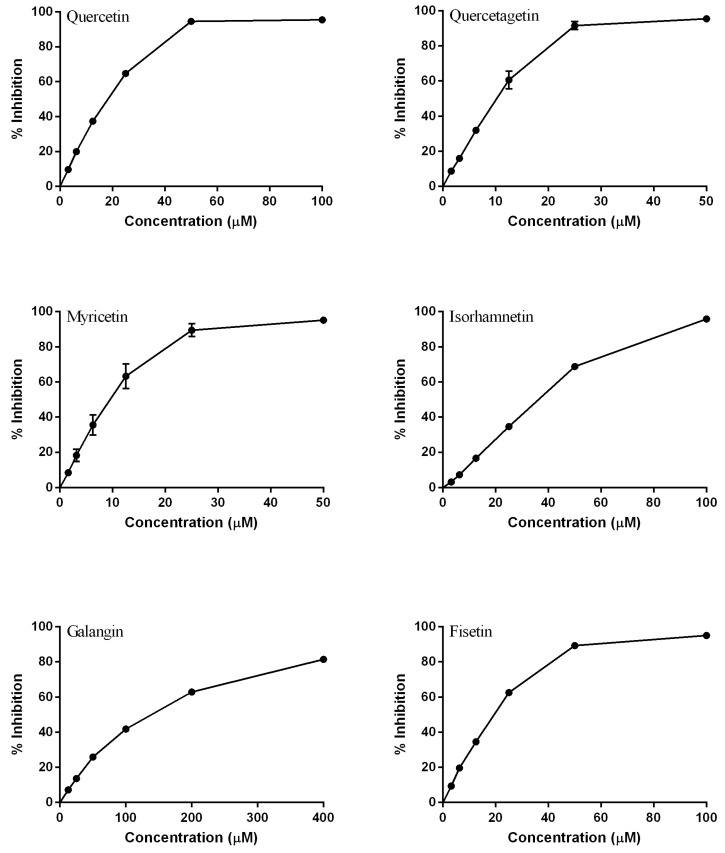
Dose-response curves of the tested flavonols (mean ± SEM of three determinations).

**Figure 2:**
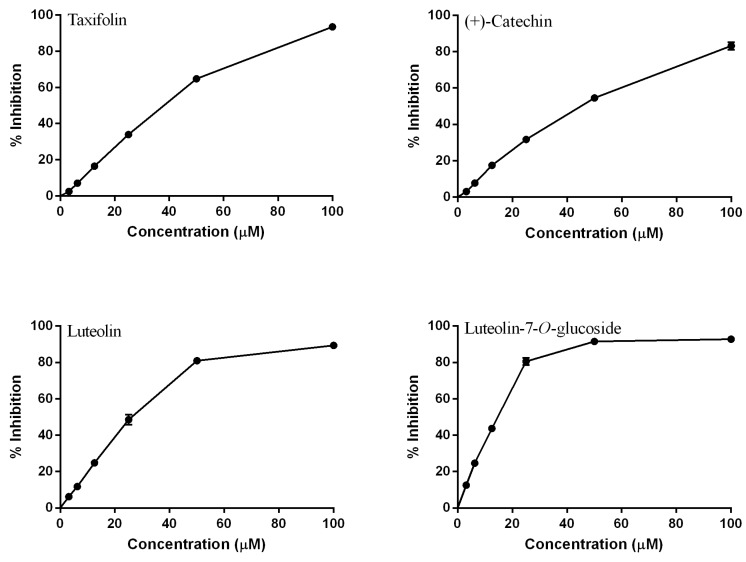
Dose-response curves of tested flavonoids from other classes (mean ± SEM of three determinations).

The structures of the studied flavonoids are presented in Table 1. A concentration dependent effect was observed for all of them under the assay conditions (Figure 1 and Figure 2). Quercetin, recognized as a reference compound, contains all the above mentioned features for antioxidant capacity [25] and showed an IC_50_ of 18.30 ± 0.14 µM (Figure 1). In order to establish structure-antioxidant activity relationships, other flavonoids, with few structural changes relatively to quercetin, were examined.

#### 2.1.1. Modifications in A Ring

To evaluate the importance of hydroxyl groups in the A ring, quercetagetin and fisetin (Table 1) were analysed. The presence of a pyrogallol A ring increased the antioxidant activity, as observed with quercetagetin (IC_50_ to 10.41 ± 0.93 µM, *p* < 0.05) (Figure 1). On the other hand, the hydroxyl group at C5 does not seem to be really important for the DPPH*^•^* scavenging activity. Fisetin, lacking this substituent, presented an IC_50_ of 19.37 ± 0.73 (Figure 1), which is not significantly different from that obtained with quercetin (*p* > 0.05).

#### 2.1.2. Modifications in the B Ring

As it was observed with the A ring, the presence of an additional hydroxyl group (pyrogallol B ring) in myricetin decreased the IC_50_ to 9.77 ± 1.52, *p* < 0.05 (Figure 1). Thus, by increasing the hydroxylation pattern in the B ring, the ability of the flavonoid to scavenge DPPH radical was also increased. Comparing quercetagetin with myricetin, no significant differences were verified between the presence of the pyrogallol in the A or B rings, respectively (*p* > 0.05).

Galangin and isorhamnetin were analysed to clarify the effect of the *o*-dihydroxy substitution in the B ring. As referred above, the catecholic B ring is important for the antioxidant activity, which is consistent with our results: the methoxylation of C3′ turned isorhamnetin less active than quercetin (IC_50_ of 36.26 ± 0.76 µM, *p* < 0.01) (Figure 1), which is in accordance with Rice-Evans *et al.* [6]; the lack of hydroxyl groups in the B ring, as occurs in galangin, had a more negative effect, as demonstrated the IC_50_ value found for this compound (138.80 ± 3.44 µM, *p* < 0.001) (Figure 1).

#### 2.1.3. Modifications in C Ring

The importance of the C2=C3 double bond to the antioxidant capacity was observed when taxifolin, lacking this feature, was tested. This compound displayed an IC_50_ of 37.97 ± 0.13 (Figure 2), which is c.a. 2-fold higher than that of quercetin (*p* < 0.001).

By comparing taxifolin with (+)-catechin, the importance of the 4-oxo group could be evaluated. The presence of this group did not affect the antioxidant activity: (+)-catechin showed an IC_50_ of 45.20 ± 2.01, *p* > 0.05 (Figure 2), which is not significantly different than that of taxifolin. Analyzing the C2=C3 double bond in conjugation with a 4-oxo group, by comparing (+)-catechin with quercetin, significant differences were found (*p* < 0.01), which confirmed the importance of this conjugation as described by other authors [6].

As for the role of the 3-OH group in the C ring, luteolin displayed an IC_50_ of 26.43 ± 1.78 (Figure 2), which is c.a. 1.5-fold higher than that of quercetin (*p* < 0.05). This result is consistent with other works reporting that this group is really important to the antioxidant activity [26].

Previous studies [27,28] reported that aglycones are more potent antioxidants than their corresponding glycosides. This observation is based on the presence of glycosyl residues at C3 of the C ring. However, our findings indicated that the glycosylation of luteolin at C7 of the A ring increased its capacity to scavenge DPPH radical (IC_50_ of 14.66 ± 0.46, *p* < 0.05) (Figure 2). Other researchers observed the same behaviour when these two compounds were tested against DPPH^•^ [29].

### 2.2. DPPH^•^ Scavenging Activity of Flavonoids in Mixtures with GSH

Additive effect is not a simple arithmetic sum of two (or more) compounds. If compounds A and B each inhibit 30%, then the additive effect is not 60%, because if A and B each inhibit 60%, the combined additive effect cannot be 120%. For this reason the simulation formula presented in the Experimental section was applied. Chou and Talalay [30] called it the fractional product method and it will never lead to a combination effect exceeding 100% inhibition. Those authors indicated that Webb’s method [31] is valid only when both compounds have hyperbolic curves, as happens with the compounds tested. If A and B each inhibit 60%, than it is oversimplification to say that the additive effect is 84% inhibition [31].

The antioxidant activity of flavonoids and their interaction with GSH *in vitro* depends upon the arrangement of functional groups about the nuclear structure. Based on the quinone/quinone methide isomerization chemistry involved in the formation of the A ring type glutathionyl adducts from quercetin *o*-quinone/quinone methide, it can be postulated that especially the presence of the C2=C3 double bond, the 3-OH group, the 4-oxo moiety, and the 5-OH and/or 7-OH groups are required for efficient quinone methide formation and GSH adduct formation in the A ring instead of in the B ring [32].

**Figure 3:**
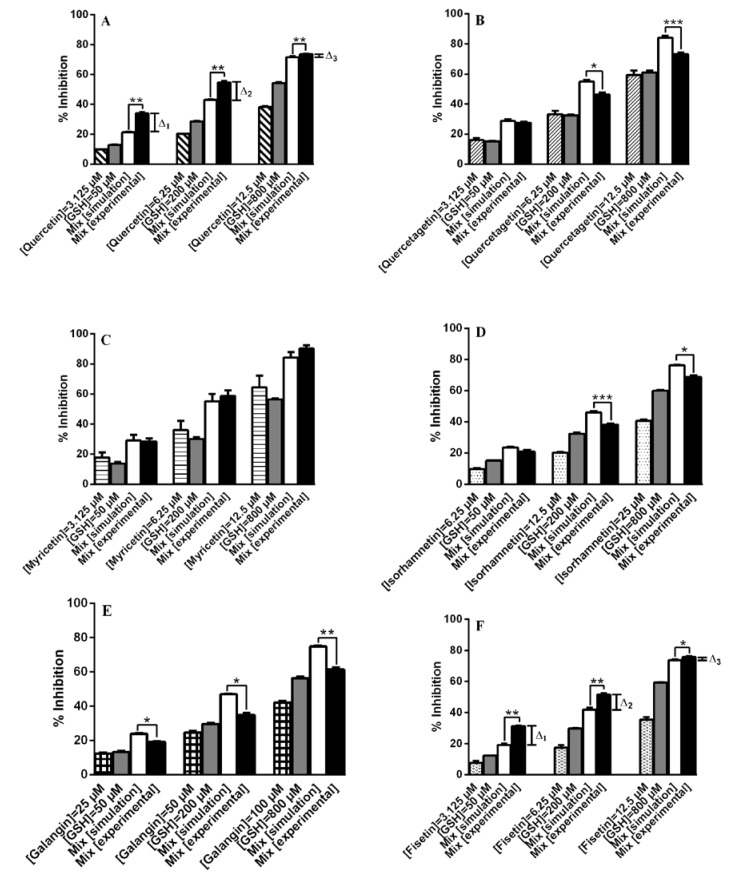
Antioxidant activity of binary mixtures of six flavonols ((**A**) Quercetin; (**B**) Quercetagetin; (**C**) Myricetin; (**D**) Isorhamnetin; (**E**) Galangin; (**F**) Fisetin) with GSH (mean ± SEM of three determinations). * *p* < 0.05, ** *p* < 0.01, *** *p* < 0.001.

**Figure 4:**
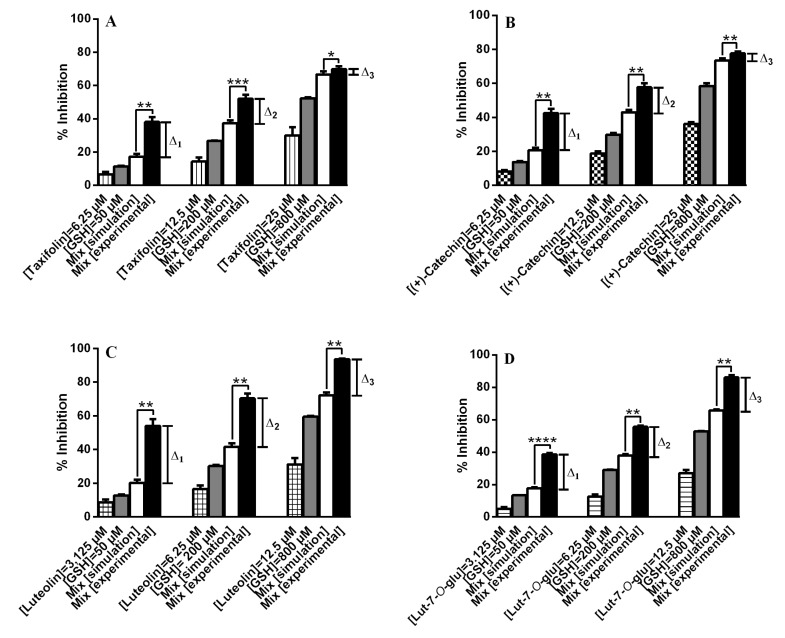
Antioxidant activity of binary mixtures of other classes of flavonoids ((**A**) Taxifolin; (**B**) (+)-Catechin; (**C**) Luteolin; (**D**) Luteolin-7-*O*-glucoside) with GSH (mean ± SEM of three determinations). * *p* < 0.05, ** *p* < 0.01, *** *p* < 0.001, **** *p* < 0.0001.

**Scheme 1:**
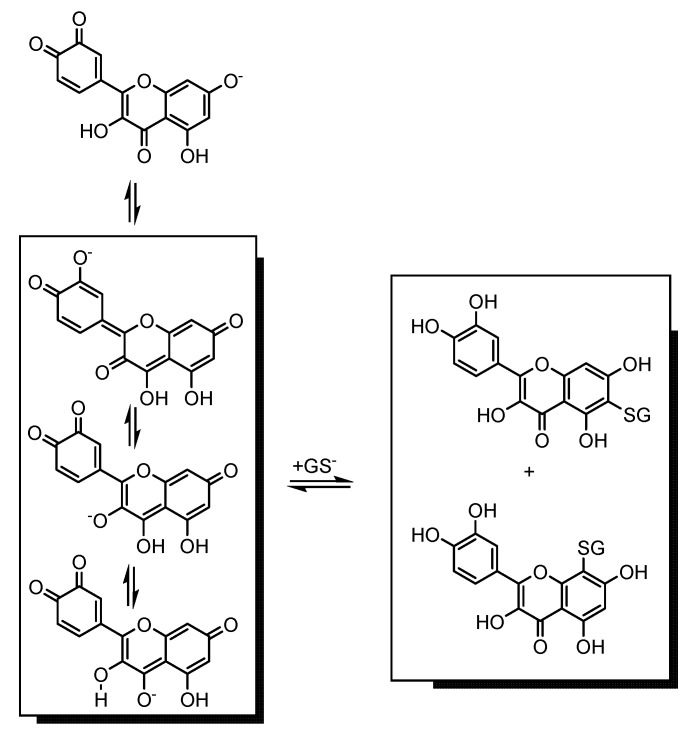
Representation of the mechanism for the formation of the glutathionyl adducts of quercetin quinone/quinone methide at neutral pH.

Among the flavonoids tested, those having a catechol group in the B ring, namely quercetin, fisetin, luteolin, luteolin-7-*O*-glucoside, taxifolin, and (+)-catechin (Table 1), showed synergistic effects with glutathione (Figure 3 and Figure 4). An exception was noted with quercetagetin (Figure 3B) which presents one additional hydroxyl group at C6 of the A ring compared to quercetin. This hydroxyl seems to be really important to its behaviour in mixtures with GSH, because the insertion of this group in quercetin to form quercetagetin, although it increases the activity of the isolated compound (*p* < 0.05), it makes the compound an antagonist of GSH (Figure 3A,B). On the other hand, myricetin with an additional OH group at C5′ of the B ring showed an additive effect with GSH (*p* ≥ 0.05) (Figure 3C). Quercetagetin and myricetin with a pyrogallol configuration in one of the rings can auto-oxidize and form superoxide radical (O_2_^•^^−^) [33]. Like our DPPH^•^ system, at neutral pH, GSH is partially present in its highly nucleophilic thiolate form, and the quercetin *o*-quinone loses its most acidic proton at 7-OH, followed by an efficient mesomeric equilibrium of the quercetin *o*-quinone monoanion with its corresponding quinone methide isomers. Quinone methide formation in the A ring is thus favoured, which gives rise to glutathionyl adduct formation in the A ring, leading to 6- and 8-glutathionyl quercetin adducts [34,35] (Scheme 1). Thus, the presence of the hydroxyl group at C6 of quercetagetin can create a steric hindrance to form active adducts with GSH and so being not able to offset the generation of O_2_^•−^ from auto-oxidation processes. On the other hand, it seems that myricetin, which presents the same structure in the A ring, can form the same adducts as quercetin and this can compensate O_2_^•−^ formation. This may explain why myricetin showed an additive effect.

To study the effect of methoxylation, isorhamnetin was analysed. The mixtures of this compound with GSH showed an antagonistic behaviour (*p <* 0.05) (Figure 3D). According to van der Woude *et al*. catechol-*O*-methylated metabolites of quercetin form covalent adducts with glutathione. These covalent adducts are formed in the A ring (6-glutathionyl 3′-*O*-methylquercetin and 8-glutathionyl 3′-*O*-methylquercetin) [36]. Thus, the formation of these adducts consumes GSH, but as they do not have the B ring catechol free, consequently the inhibition is lower than that of the simulation. A similar situation occurs with galangin, which has a benzene B ring (*p* < 0.05) (Figure 3E).

**Scheme 2:**
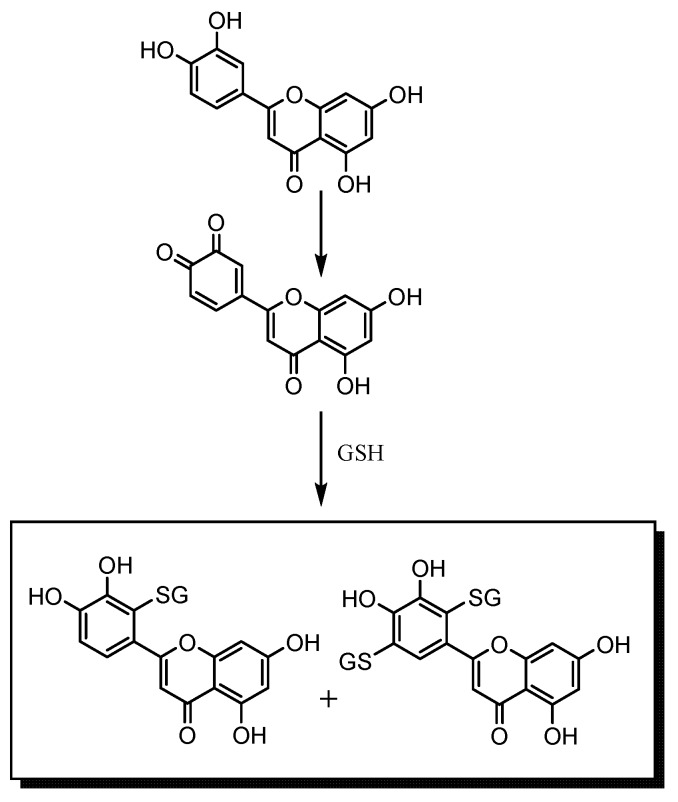
Representation of the mechanism for the formation of the mono- and diglutathionyl adducts of luteolin.

With taxifolin and luteolin the absence of the C2=C3 double bond and the 3-OH group, respectively, hamper the quinone methide isomerization of their *o*-quinone and deprotonation states, and, thus, the GSH/GS^−^ addition preferentially occurs in the B ring. The formation of 2′,5′-diglutathionyl adducts for taxifolin and luteolin starts to occur when the concentration and reactivity of the 2′-monoglutathionyl adduct starts to compete as substrate with the parent flavonoid, a process depending on its concentration and ionization potential (Scheme 2) [37]. Comparing the isolated activity of quercetin with that of luteolin, no significant differences were observed for the three concentrations tested (3.125, 6.25 and 12.5 µM, *p* ≥ 0.05). On the other hand, comparing the variation between experimental and simulation results of the same concentrations of quercetin (Δ_1_ = 12.64 ± 0.85, Δ_2_ = 11.63 ± 1.25, Δ_3_ = 1.95 ± 0.09) and luteolin (Δ_1_ = 33.74 ± 2.55, Δ_2_ = 28.75 ± 2.08, Δ_3_ = 21.42 ± 1.58), significant differences were observed (*p* < 0.05) (Figure 3A and Figure 4C). With these results it seems possible to expect the presence of this 2’-monoglutathionyl adduct and diglutathionyl adducts in the B ring to favour more the antioxidant activity than 6-glutathionyl and 8-glutathionyl adducts in the A ring.

To observe the impact of the glycosylation, luteolin-7-*O*-glucoside was also studied. For three concentrations tested (3.125, 6.25 and 12.5 µM) no significant differences were found between the aglycone, luteolin, and its glycoside (*p* ≥ 0.05). However, at concentrations of 3.125 and 6.25 µM, the mixtures with the aglycone form are more interesting in terms of antioxidant activity (*p* < 0.05): Δ_1_ = 33.74 ± 2.55, Δ_2_ = 28.75 ± 2.08, Δ_3_=21.41 ± 1.58 (Figure 4C) and Δ_1_ = 20.97 ± 0.13, Δ_2_ = 17.69 ± 1.03, Δ_3_ = 20.42 ± 1.74 (Figure 4D) for luteolin and its glucoside, respectively.

With fisetin, the lack of the 5-OH group in relation to quercetin did not affect the antioxidant activity at concentrations tested (*p* ≥ 0.05). An interesting aspect is that these two compounds also exhibited the same behaviour when in mixture with GSH, as no significant differences of the variation between experimental and simulation results were observed (*p* ≥ 0.05): the Δ values found for fisetin were Δ_1_ = 12.26 ± 0.76, Δ_2_ = 9.63 ± 0.31, Δ_3_ = 2.07 ± 0.33 (Figure 3F). These results seem to suggest that fisetin and quercetin form the same adducts in C6 and C8 or originate different adducts with the same antioxidant activity.

Taxifolin and (+)-catechin presented synergistic effect in mixtures with GSH (*p* < 0.05) (Figure 4A,B). Moridani *et al.* [38] verified the formation of mono- and biglutathionyl adducts in 2′ and 5′ in B ring of (+)-catechin, as it happened with taxifolin. The same variation observed between experimental and simulation results with these two compounds (Δ_1_ = 20.88 ± 1.25, Δ_2_ = 14.89 ± 0.41, Δ3 = 3.17 ± 0.45 for taxifolin (Figure 4A) and Δ1 = 21.68 ± 1.89, Δ2 = 14.74 ± 0.85, Δ3 = 4.22 ± 0.35 for (+)-catechin (Figure 4B) perhaps may be explained by the formation of the same glutathionyl adducts, that consequently could have the same activity.

## 3. Experimental

### 3.1. Reagents

GSH, DPPH^•^, dimethyl sulfoxide (DMSO), and quercetin, were from Sigma–Aldrich (St. Louis, MO, USA). Quercetagetin, luteolin, luteolin-7-*O*-glucoside, isorhamnetin, galangin, fisetin, myricetin, (+)-catechin, and taxifolin were from Extrasynthese (Genay, France). Methanol was from Atom Scientific (Manchester, UK).

### 3.2. Compounds Solutions and Binary Mixtures

Stock solutions (1.8 mg/mL) of the tested flavonoids (Table 1) were prepared in methanol, excepting for luteolin-7-*O*-glucoside and isorhamnetin that were dissolved in methanol with 10% DMSO (no solvent interference). Glutathione stock solution (4.20 mg/mL) was prepared in water.

The binary mixtures were prepared with three different concentrations of GSH (50, 200 and 800 μM), corresponding to low, intermediate and high percentage of radical scavenging, as obtained from a dose-response curve. The flavonoids concentrations used in the mixtures were determined from preliminary trials, which indicated the upper and lower limits that were necessary to visualize the effect without complete reduction of DPPH^•^.

### 3.3. Antioxidant Activity

#### 3.3.1. DPPH^•^ Scavenging Activity

The disappearance of DPPH^•^ was monitored spectrophotometrically at 515 nm on a Multiskan Ascent plate reader (Thermo, Electron Corporation, Shanghai, China), following a described procedure [39]. The reaction in the sample wells consisted of 25 μL of tested solution (flavonoid, GSH or binary mixture) and 200 μL of 150 μM DPPH^•^ dissolved in methanol. To guarantee the completeness of reactions the absorbance was measured every 5 min (data not shown). A 30 min period starting with the addition of the radical revealed to be sufficient for complete reaction and the absorbance recorded after this time was used in all calculations.

In order to compare the activity of isolated compounds, half-maximal inhibitory concentration (IC_50_) was calculated. Three individual experiments were performed, in which each concentration was tested in triplicate.

#### 3.3.2. Determination of Mixture Effect (Simulation)

To evaluate the behaviour of the flavonoids when combined with GSH (synergistic or antagonistic effects), experimental results were compared with those of a simulation, following Webb [30]:

[Simulation (%) = 1 − ((1 − Inhibition _GSH_) × (1 − Inhibition _flavonoid_)) × 100]

The effect was considered to be additive when no significant differences were observed between experimental and simulation results. On the other hand, synergistic and antagonistic effects correspond to the existence of significant differences between experimental and simulation results: synergism was considered to occur when the experimental % inhibition was higher than that of the simulation, while an antagonist effect corresponded to lower experimental % inhibition compared to simulation results.

### 3.4. Statistical Analysis

Results are shown as mean (±SEM) of three determinations. Experimental and simulation results were submitted to paired *t*-test (GraphPad Prism 6 Software, Inc, San Diego, CA, USA) and differences at *p* < 0.05 were considered statistically significant. The variation (Δ) between simulation and experimental results was also determined and submitted to paired *t*-test.

## 4. Conclusions

The results obtained in the present study revealed that flavonoid intake through supplementation and/or diet may not always be favourable due to their different interactions with the major endogenous antioxidant (GSH). Although all tested flavonoids have antioxidant activity when isolated, galangin and ishoramnetin antagonized the effect of GSH, decreasing the endogenous antioxidant capacity. The presence of a catechol group in the B ring was demonstrated to be essential for synergisms with GSH, except when an OH group at C6 is also present (like in quercetagetin). In addition, the adducts formed with GSH in the B ring are more active than those in the A ring. Hereupon, as oxidative processes are involved in many diseases, the observed interactions are also very important for human health. Despite the anti-radical capacity of the tested compounds and the effects observed *in vitro* in the presence of the most important endogenous antioxidant, the potential hazards/benefits observed *in vivo* will depend on their bioavailability and biotransformation. 

## References

[1] Thomas J.A., Poland B., Honzatko R. (1995). Protein sulfhydryls and their role in the antioxidant function of protein S-thiolation. Arch. Biochem. Biophys..

[2] Huang K.P., Huang F.L. (2002). Glutathionylation of proteins by glutathione disulfide S-oxide. Biochem. Pharmacol..

[3] Hayes J.D., Pulford D.J. (1995). The glutathione S-transferase supergene family: Regulation of GST and the contribution of the isoenzymes to cancer chemoprotection and drug resistance. Crit. Rev. Biochem. Mol. Biol..

[4] Wild A.C., Mulcahy R.T. (2000). Regulation of γ-glutamylcysteine synthetase subunit gene expression: Insights into transcriptional control of antioxidant defenses. Free Radic. Res..

[5] Rice-Evans C.A., Miller N.J., Bolwell P.G., Bramley P.M., Pridham J.B. (1995). The relative antioxidant activities of plant-derived polyphenolic flavonoids. Free Radic. Res..

[6] Rice-Evans C.A., Miller N.J., Paganga G. (1996). Structure-antioxidant activity relationships of flavonoids and phenolic acids. Free Radic. Biol. Med..

[7] Milella L., Caruso M., Galgano F., Favati F., Padula M.C., Martelli G. (2011). Role of the cultivar in choosing Clementine fruits with a high level of health-promoting compounds. J. Agric. Food Chem..

[8] Walle T. (2004). Absorption and metabolism of flavonoids. Free Radic. Biol. Med..

[9] Gaikwad P., Barik A., Priyadarsini K.I., Rao B.S.M. (2010). Antioxidant activities of phenols in different solvents using DPPH assay. Res. Chem. Intermed..

[10] Beecher C.W.W. (1994). Cancer preventive properties of varieties of *Brassica oleracea*—A review. Am. J. Clin. Nutr..

[11] Van Poppel G., Verhoeven D.T.H., Verhagen H., Goldbohm R.A. (1999). *Brassica* vegetables and cancer prevention—Epidemiology and mechanisms. Adv. Nutr. Cancer.

[12] Millic B.L., Diljas S.M., Canadanovic-Brunet J.M. (1998). Antioxidative activity of phenolic compounds on the metal-ion breakdown of lipid peroxidation system. Food Chem..

[13] Karadag A., Ozcelik B., Saner S. (2009). Review of methods to determine antioxidant capacities. Food Anal. Methods.

[14] Menezes J.C., Kamat S.P., Cavaleiro J.A., Gaspar A., Garrido J., Borges F. (2011). Synthesis and antioxidant activity of long chain alkyl hydroxycinnamates. Eur. J. Med. Chem..

[15] Arnao M.B. (2000). Some methodological problems in the determination of antioxidant activity using chromogen radicals: A practical case. Trends Food Sci. Technol..

[16] Villaño D., Fernández-Pachon M.S., Troncoso A.M., Garcia-Parrilla M.C. (2004). The antioxidant activity of wines determined by the ABTS^•+^ method: Influence of sample dilution and time. Talanta.

[17] Marais J.P.J., Deavours B., Dixon R.A., Ferreira D., Grotewold E. (2007). The Stereochemistry of Flavonoids. The Science of Flavonoids.

[18] Boots A.W., Kubben N., Haenen G.R., Bast A. (2003). Oxidized quercetin reacts with thiols rather than with ascorbate: Implication for quercetin supplementation. Biochem. Biophys. Res. Commun..

[19] Kessler M., Ubeaud G., Jung L. (2003). Anti- and pro-oxidant activity of rutin and quercetin derivatives. J. Pharm. Pharmacol..

[20] Yao L.H., Jiang Y.M., Shi J., Tomás-Barberán F.A., Datta N., Singanusong R., Chen S.S. (2004). Flavonoids in food and their health benefits. Plant Foods Hum. Nutr..

[21] Heimann W., Reiff F. (1953). Beziehung zwischen chemischer konstitution und antioxygener wirkung bei flavonolen. Fette Seifen Anstr-Mittel..

[22] Bors W., Heller W., Michel C., Saran M. (1990). Flavonoids as antioxidants: Determination of radical scavenging efficiencies. Methods Enzymol..

[23] Sichel G., Corsaro C., Scalia M., di Bilio A.J., Bonomo R.P. (1991). *In vitro* scavenger activity of some flavonoids and melanins against O_2_^•−^. Free Radic. Biol. Med..

[24] Rastija V., Medić-Sarić M. (2009). QSAR study of antioxidant activity of wine polyphenols. Eur. J. Med. Chem..

[25] Harborne J.B., Mabry T.J., Mabry H. (1975). The Flavonoids.

[26] Burda S., Oleszek W. (2001). Antioxidant and antiradical activities of flavonoids. J. Agric. Food Chem..

[27] Ratty A.K., Das N.P. (1988). Effects of flavonoids on nonenzymatic lipid peroxidation: Structure-activity relationship. Biochem. Med. Metab. Biol..

[28] Matthiesen L., Malterud K.E., Sund R.B. (1997). Hydrogen bond formation as basis for radical scavenging activity: A structure-activity study of *C*-methylated dihydrochalcones from *Myrica gale* and structurally related acetophenones. Free Radic. Biol. Med..

[29] Wu M.J., Huang C.L., Lian T.W., Kou M.C., Wang L. (2005). Antioxidant activity of *Glossogyne tenuifolia*. J. Agric. Food Chem..

[30] Chou T.C., Talalay P. (1984). Quantitative analysis of dose-effect relationships: The combined effects of multiple drugs or enzyme inhibitors. Adv. Enzyme Regul..

[31] Webb J.L. (1963). Effect of More than One Inhibitor. Enzyme and Metabolic Inhibitors.

[32] Awad H.M., Boersma M.G., Boeren S., van Bladeren P.J., Vervoort J., Rietjens I.M. (2002). The regioselectivity of glutathione adduct formation with flavonoid quinone/quinone methides is pH-dependent. Chem. Res. Toxicol..

[33] Hodnick W.F., Kung F.S., Roettger W.J., Bohmont C.W., Pardini R.S. (1986). Inhibition of mitochondrial respiration and production of toxic oxygen radicals by flavonoids: A structure-activity study. Biochem. Pharmacol..

[34] Frey-Schroder G., Barz W. (1979). Isolation and characterization of flavonol converting enzymes from *Mentha piperita* plants and from *Mentha arvensis* cell suspension cultures. Z. Naturforsch..

[35] Dangles O., Fargeix G., Dufour C. (1999). One-electron oxidation of quercetin derivatives in protic and non protic media. J. Chem. Soc. Perkin Trans..

[36] Van der Woude H., Boersma M.G., Alink G.M., Vervoort J., Rietjens I.M. (2006). Consequences of quercetin methylation for its covalent glutathione and DNA adduct formation. Chem. Biol. Interact..

[37] Awad H.M., Boersma M.G., Boeren S., van Bladeren P.J., Vervoort J., Rietjens I.M.C.M. (2001). Structure-activity study on the quinone/quinone methide chemistry of flavonoids. Chem. Res. Toxicol..

[38] Moridani M.Y., Scobie H., Salehi P., O’Brien P.J. (2001). Catechin metabolism: Glutathione conjugate formation catalyzed by tyrosinase, peroxidase, and cytochrome p450. Chem. Res. Toxicol..

[39] Vrchovská V., Sousa C., Valentão P., Ferreres F., Pereira J.A., Seabra R.M., Andrade P.B. (2006). Antioxidative properties of tronchuda cabbage (*Brassica oleracea* L. var. *costata* DC) external leaves against DPPH^•^, superoxide radical, hydroxyl radical and hypochlorous acid. Food Chem..

